# Analysis of the Efficacy and Safety of Weekly Calcifediol 100 µg in Vitamin D Deficient Patients

**DOI:** 10.3390/jcm14092976

**Published:** 2025-04-25

**Authors:** Jose Luis Pérez Castrillón, Esteban Jódar-Gimeno, Koldobika Molina, Aintzane García-Bea, Cristina Martínez Ostalé, Inmaculada Gilaberte

**Affiliations:** 1 Department of Internal Medicine, Río Hortega University Hospital, 47012 Valladolid, Spain; 2 Department of Endocrinology and Nutrition, Quirónsalud Madrid University Hospital, 28223 Madrid, Spain; 3 Clinical Research Department, FAES FARMA, 48940 Leioa, Spain; 4 Medical Affairs Department, FAES FARMA, 48940 Leioa, Spain

**Keywords:** vitamin D, deficiency, hypovitaminosis, supplementation, calcifediol, 25(OH)D, randomized trial, phase II/III

## Abstract

**Background/Objectives**: Adequate vitamin D levels are critical for overall health, yet vitamin D deficiency remains prevalent. This study aims to evaluate the efficacy and safety of a standardized weekly supplementation regimen of 100 μg calcifediol for patients with varying degrees of vitamin D deficiency. **Methods**: A *post hoc* pool analysis was conducted from a randomized, double-blind, placebo-controlled, multicenter, two-cohort trial. Cohort 1 included vitamin D mild deficiency patients (25(OH)D levels > 10 < 20 ng/mL) and Cohort 2 severe deficiency patients (25(OH)D levels ≤ 10 ng/mL). As both had placebo and weekly calcifediol 100 μg arms (ratio 1:2), a pooled analysis of safety and efficacy was conducted. The primary outcome was the percentage of subjects achieving 25(OH)D levels ≥ 20 ng/mL and/or ≥30 ng/mL at various time points. **Results**: A total of 401 participants across both cohorts were included in the analysis, 130 who received a placebo and 271 calcifediol 100 µg weekly. By week 52, 94.5% of individuals in the calcifediol group achieved 25(OH)D levels ≥ 20 ng/mL, compared to 25.3% in the placebo group (*p* < 0.0001). At this same week, 80.5% of subjects in the calcifediol group, but none in the placebo group (*p* < 0.0001), had 25(OH)D levels ≥ 30 ng/mL. The mean 25(OH)D level plateaued around 40.7 ng/mL from weeks 16 to 52. The frequency of treatment-emergent adverse events was similar in both groups, placebo and calcifediol. **Conclusions**: Weekly supplementation of 100 μg calcifediol effectively restores vitamin D levels in individuals with both mild and severe deficiencies, demonstrating a favourable safety profile.

## 1. Introduction

Besides playing important roles in calcium homeostasis and bone mineralization, vitamin D (VD) has recently attracted significant attention due to its pleiotropic effects on the immune system, cardiovascular health, cancer prevention and metabolic diseases [1,2,3]. This association can be partly explained by the presence of VD receptors in more than 30 biological tissues [4].

The bioavailability of vitamin D is generally evaluated through its major circulating metabolite, 25-hydroxyvitamin D, 25(OH)D, which has a circulating half-life of 2–3 weeks, serving as a stable biomarker of systemic VD status [5]. Deficient levels of 25(OH)D, typically defined as concentrations below 20 ng/mL, represent a global concern, affecting individuals across all age groups, from children to the elderly [6]. In some regions, such as Europe, a prevalence of over 50% has been reported [7], highlighting the need for efficient treatment strategies to address vitamin D deficiency.

Among the available therapeutic options for the treatment of vitamin D deficiency, calcifediol [25(OH)D_3_] has been shown to be the fastest and most potent at increasing 25(OH)D levels, showing a good safety profile [8,9], making it an excellent option for preventing and treating hypovitaminosis D. In Spain, calcifediol has been used for over 40 years in the management of vitamin D deficiency, with its long history of clinical use supporting its efficacy and safety and contributing to its more recent adoption in numerous other European and Latin American countries. According to Jódar et al. [10] calcifediol is suitable for all patients with vitamin D deficiency and may present advantages over vitamin D3 (cholecalciferol) in specific populations, such as those with obesity, liver disease, malabsorption issues, or in cases where a rapid increase in 25(OH)D concentrations is required. These advantages could be further enhanced by implementing a weekly dosing regimen, potentially offering improved adherence and consistency compared to the current daily or monthly regimens.

In two previously published articles, the results of the efficacy of three new formulations of weekly calcifediol was investigated in two distinct cohorts: Cohort 1, comprising patients with moderate vitamin D deficiency (baseline 25(OH)D levels: 10 to 20 ng/mL), and Cohort 2, including patients with severe deficiency (baseline 25(OH)D levels: ≤10 ng/mL) [11,12]. Weekly treatment of Cohort 1 with 75 µg or 100 µg and Cohort 2 with 100 µg or 125 µg of calcifediol was demonstrated to be highly efficient in restoring 25(OH)D levels. This raises the question of whether a 100 µg dose could be universally effective across all levels of vitamin D deficiency regardless of basal levels.

To address this question, we conducted a *post hoc* analysis pooling subjects who received a 100 µg dose of calcifediol from both cohorts and compared them to a pooled placebo group. This approach aimed to evaluate the generalizability of weekly formulations across different baseline deficiency levels and, consequently, explore the potential for a simplified, standardized dosing regimen in clinical practice.

## 2. Materials and Methods

### 2.1. Participants

This is a *post hoc* study carried out from a randomized, double-blind, two-cohorts, double-dummy, multicentre, dose-ranging, phase II/III clinical trial. Subjects were allocated to 2 cohorts based on their 25(OH)D baseline level at visit 1: Cohort 1 (25(OH)D levels > 10 to <20 ng/mL) and Cohort 2 (25(OH)D levels ≤ 10 ng/mL). This *post hoc* study was performed to evaluate individuals who received a placebo or 100 µg calcifediol once a week in both cohorts as a pool. Participants of the study were male or female ≥ 18 years of age and signed informed consent forms. Subjects were excluded if they were receiving any previous treatment with calcifediol, vitamin D analogues, vitamin complexes or vitamin D supplements within the last week before screening or planned during the clinical study or if taking drugs that could modify vitamin D levels (e.g., some antibiotics, antiretrovirals, long-term corticosteroids). Conditions that could potentially influence calcifediol absorption and metabolism, such as severe renal impairment, malabsorption syndromes, and liver or biliary dysfunction, were included as exclusion criteria to ensure the accuracy of the study outcomes. Complete eligibility criteria are defined in Supplemental Table S1.

**Figure 1:**
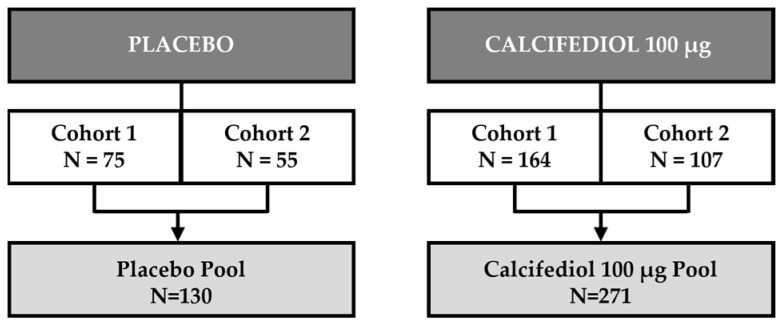
Analysis population. Subjects included in the Placebo and Calcifediol 100 µg pools from cohort 1 (25(OH)D levels > 10 to <20 ng/mL) or Cohort 2 (25(OH)D levels ≤ 10 ng/mL).

In this analysis, were included 239 subjects with mild vitamin D deficiency from cohort 1 and 162 subjects with severe vitamin D deficiency from cohort 2, of whom 130 comprised the placebo pool and 271 the pool treated with calcifediol 100 µg (Figure 1).

### 2.2. Methodology

The study was conducted in European countries (Bulgaria, Czech Republic, Spain, France, Italy, Serbia and Slovakia) from 28 December 2020 to 25 April 2023. The protocol was approved by an Independent Ethics Committee prior to each site initiation. The study adhered to the ethical principles outlined in the Declaration of Helsinki, and all participants were signed with written informed consent prior to enrolment in the study. The study was registered at Clinicaltrials.gov (NCT04735926) and with EudraCT number 2020-001099-14.

After screening, subjects were randomized to placebo or calcifediol 100 µg in a 1:2 ratio in both cohorts independently. The treatment phase started after randomization and continued for 52 weeks, with a subsequent 30-day follow-up. The subjects had to attend 7 on-site visits and 1 telephone follow-up visit. Calcifediol soft gelatin capsules (SGC) of 100 µg were provided by FAES FARMA SA. Placebo SGC contained the same excipients and was also manufactured by FAES FARMA SA. Calcifediol and placebo capsules were identical in size, colour, taste and appearance. They had to be administered orally once per week on Sunday morning with or without a meal, swallowed whole and could be taken with water, milk or juice. Subjects with 25(OH)D concentration ≤ 10ng/mL after 16, 24 or 32 weeks of treatment additionally received daily cholecalciferol 800 IU as rescue medication.

The primary objective of this *post hoc* analysis was to determine and compare the percentage of subjects with 25(OH)D levels ≥ 20 ng/mL and/or 25(OH)D ≥ 30 ng/mL at 4, 16, 24, 32 and 52 weeks of treatment. Secondary objectives included the evaluation of 25(OH)D levels over time and the safety profile across the 52 weeks of treatment. In addition to treatment-emergent adverse events (TEAEs) reporting, safety assessments included ECG recordings, vital constants records (systolic blood pressure, diastolic blood pressure, heart rate and body temperature) and laboratory safety tests (hematology and biochemistry). The variations observed in these assessments were evaluated in terms of clinically relevant changes. The frequency of TEAEs is presented as the percentage of subjects in each treatment group who experienced any serious or related TEAE. Additionally, changes in bone mineral parameters were analyzed, and data was presented as the percentage of patients with abnormal levels at baseline and in week 52.

### 2.3. Analyses

Blood samples were analysed in a central laboratory (LKF—Laboratorium für Klinische Forschung GmbH). 25(OH)D serum and PTH concentrations were determined using an immunoassay system (ECLIA). Concentrations of total serum calcium (tCa) were obtained in the same central laboratory using an automated analyser. The samples were collected at the screening visit and after 4, 16, 24, 32, and 52 weeks.

### 2.4. Statistical Analysis

All patients who received at least one investigational medicine were included in the safety reporting (Safety Population). Among them, subjects with at least one postbaseline assessment of 25(OH)D levels, excluding those who received rescue medication, were considered for *post hoc* statistical analyses (Full Analysis Population). Continuous data of each treatment group were summarized using descriptive statistics. For statistical significance, a *p*-value < 0.05 was considered appropriate. SAS^®^ (version 9.4, SAS Institute Inc., Cary, NC, USA) was used for analyses within a validated and secure environment.

## 3. Results

**Figure 2:**
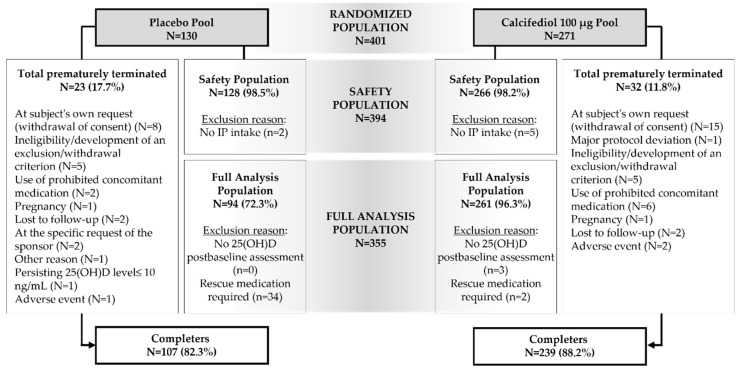
Study flow, including individuals in the placebo group and calcifediol 100 μg, the reasons for withdrawal and the number of subjects in each study population with reasons for exclusion are indicated. IP, Investigational Product.

Prior to clinical trial treatment administration some enrolled patients decline to participate and were no part of the 394 subjects conforming the Safety Population. A total of 34 subjects in the placebo pool (26.6% of total) and 2 in the calcifediol 100 µg pool (0.76%) needed rescue medication as their 25(OH)D plasma concentration was ≤10 ng/mL at weeks 16, 24, or 32, and they were excluded from statistical analysis. Three additional subjects in the calcifediol group were not included in the Full Analysis Population as they do not have any 25(OH)D assessment following treatment initiation. The whole 52 weeks study was completed by 107 individuals (82.3%) in the placebo group and 239 (88.2%) in the group with active supplement (Figure 2).

There were no relevant differences in age between groups (mean: 51.3 years in placebo and 53 years in calcifediol, respectively). Seventy-three percent (73%) were women, and around 96% were white in both groups. Overall, both groups were comparable in terms of comorbidity, age, race, and BMI (Table 1). Baseline 25(OH)D values were also similar in both pooled groups (13.2 ± 3.7 ng/mL in placebo and 12.2 ± 4.2 ng/mL in calcifediol, respectively; *p* > 0.05). The most usual ongoing medical conditions observed were hypertension (36.7% in placebo and 14.0% in calcifediol groups) and menopause (47.9% in placebo and 39.1% in calcifediol group).

### 3.1. Rate of Subjects with Vitamin D Levels ≥ 20 ng/mL and/or ≥30 ng/mL over Time

**Figure 3:**
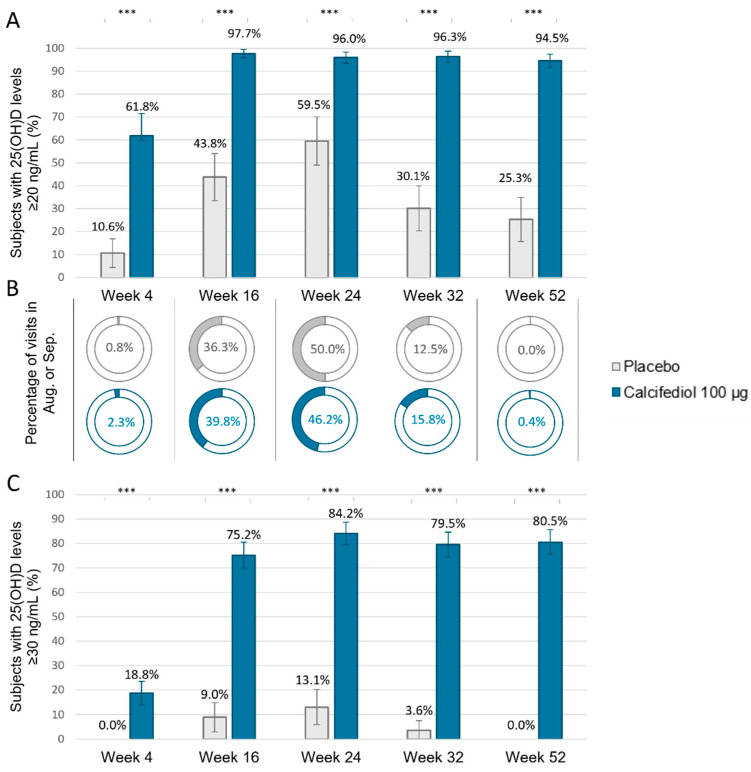
Percentage of subjects in the weekly placebo (grey; *n* = 94) and calcifediol 100 µg (blue; *n* = 261) pools of the Full Analysis Population, (**A**) subjects with 25(OH)D levels ≥ 20 ng/mL; (**B**) Percentage of subjects at the indicated time points that coincided with the months of August or September. (**C**) Percentage of subjects with 25(OH)D levels ≥ 30 ng/mL. *** *p*-value < 0.0001, obtained by two-sided comparisons of proportions. 98.75% confidence intervals (CI) are indicated by error bars.

The percentage of patients with 25(OH)D levels reaching ≥ 20 and/or ≥30 ng/mL (Figure 3) was significantly higher in the calcifediol 100 µg pool than in the placebo pool at all evaluated times after treatment initiation (*p* < 0.0001 ***). From week 16 to the end of the study, almost all calcifediol-treated subjects (>94%) had 25(OH)D values greater than 20 ng/mL, and over 75% reached target levels of ≥30 ng/mL (Figure 3). However, the highest percentage of the placebo group that reached the 25(OH)D threshold of 20 and 30 ng/mL was 59.5% and 13.1%, respectively, both at week 24. It is noteworthy that for 50% of subjects in the placebo group, week 24 coincided with visits conducted in August or September, which are known as the months with the highest endogenous vitamin D levels in the Northern Hemisphere [13,14] (Figure 1 middle panel).

### 3.2. 25(OH)D Levels over Time

**Figure 4:**
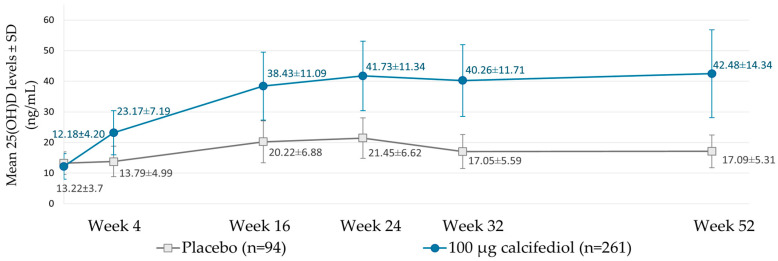
25(OH)D levels of the placebo (grey) and calcifediol 100 µg (blue) groups of the Full Analysis Population at the indicated week following the start of treatment. Data is represented as mean ± standard deviation (SD).

The evolution of 25(OH)D levels throughout the study is depicted in Figure 4. The corresponding magnitude of these changes from baseline 25(OH)D levels is represented in Supplementary Figure S1. It can be observed that during treatment with calcifediol 100 µg weekly, 25-hydroxyvitamin D levels increased rapidly, reaching its maximum of 41.73 ng/mL at week 24 and remaining stable until week 52. The differences with the placebo group were statistically significant at all study visits (*p* < 0.0001).

From week 16 to 52 of weekly calcifediol treatment, 25(OH)D levels remained with minimal fluctuations (Figure 4). Calculating the mean value observed during this stable period, weekly treatment with 100 µg calcifediol resulted in a mean *plateau* 25(OH)D level of 40.7 ng/mL.

### 3.3. Safety Results

**Figure 5:**
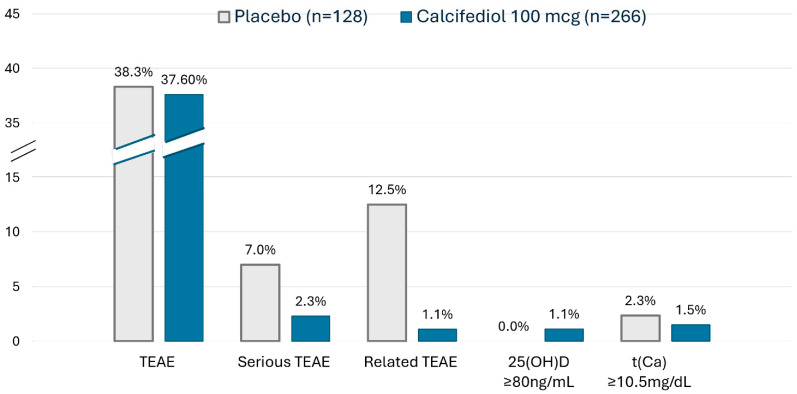
Safety Summary in the Safety Population. Percentage of subjects with any TEAE, a serious TEAE, a TEAE related to treatment, with elevated 25(OH)D levels or elevated t(Ca) levels in the placebo (grey) and calcifediol 100 µg (blue) groups. TEAE, Treatment-Emergent Adverse Event. T(Ca), total serum Calcium.

During the study, a total of 394 subjects received at least one dose of study treatments calcifediol or placebo and were evaluated for safety (Safety Population). The administration of weekly calcifediol 100 µg showed a favourable safety profile similar to placebo (Figure 5). None of the serious TEAEs, most commonly belonging to infections and infestations, was assessed as being related to treatment.

Regarding the potential toxicity of elevated concentrations of 25(OH)D, a cut-off point of ≥80 ng/mL was established in the study for discontinuation of the medication. Only three events (1.1%) of 25(OH)D levels ≥ 80 ng/mL were observed in the calcifediol group, and none were related to an adverse event. There was a higher percentage of individuals with hypercalcemia in the placebo group (2.3%, 3 subjects) than in the calcifediol group (1.5%, 4 subjects), with no observed correlation between hypercalcemia and high levels of 25(OH)D.

No relevant mean changes from baseline were observed in physical examinations, ECG records, vital signs, or haematology or biochemistry values. The highest effect of vitamin D treatment in plasma bone and mineral parameters was observed in the reduction of abnormally high parathyroid hormone levels from 20.6% to 6.5% of subjects weekly treated with 100 µg of calcifediol (Table 2).

## 4. Discussion

The present pooled analysis of vitamin D deficient patients receiving weekly 100 µg calcifediol versus placebo over 52 weeks highlights the efficacy and safety of this calcifediol formulation, regardless of 25(OH)D basal levels, positioning it as a new therapeutic alternative.

The 25(OH)D threshold necessary to obtain both skeletal and extraosseous benefits is not clearly defined. In this study, weekly administration of 100 µg of calcifediol to vitamin D deficient patients was demonstrated to restore 25(OH)D levels to the minimum target value established by the Institute of Medicine (above 20 ng/mL, [15]), and by other authors and scientific associations (above 30 ng/mL). These higher levels are especially advised for people with osteoporosis or those at significant risk of vitamin D deficiency, including the elderly [16,17,18]. After one year of treatment, 25(OH)D levels in patients with moderate and severe vitamin D deficiency who were supplemented with weekly 100 µg calcifediol reached 20 ng/mL in 94.5% of the individuals and 30 ng/mL in 80.5% of them. However, most patients achieved these threshold levels by week 4 or 16, respectively. These findings contribute to the existing literature by demonstrating that calcifediol supplementation is a safe and effective strategy, presenting several advantages over cholecalciferol. Calciferol is faster-acting, more potent, and results in more predictable increases in 25(OH)D levels, supporting its role as a preferred therapeutic strategy [8,19,20].

Compared to similar studies, our findings in a larger population with a longer follow-up align with prior research demonstrating the efficacy of calcifediol in rapidly increasing and sustaining optimal serum 25(OH)D concentrations. Jetter et al. [21] reported that serum concentrations of at least 30 ng/mL were also achieved within approximately two weeks with either a daily intake of 20 µg or a weekly intake of 140 µg calcifediol. Graeff-Armas et al. [22] investigated a similar dose, administering 15 µg/day (equivalent to 105 µg/week) to 25 participants with a higher mean baseline 25(OH)D level of 19.2 ng/mL. Similar to our study, after six months of supplementation, mean serum vitamin D values were experimented with an increment of around 30 ng/mL, with no reported adverse effects [22]. Notably, individuals also exhibited a rapid initial increase in 25(OH)D, followed by a plateau phase, further supporting the consistent and sustained response to calcifediol.

The dose-dependent effect of calcifediol on increasing 25(OH)D levels is observed when compared with findings from previous studies evaluating different doses [11,12,23,24]. Notably, Das et al. [24] reported that daily doses of 25 µg and 50 µg calcifediol (equivalent to 175 µg and 350 µg per week, respectively) resulted in higher increases in 25(OH)D concentrations reaching levels of 76.0 ng/mL and 94.3 ng/mL after 8 weeks of treatment. These higher doses demonstrated a good safety profile, further supporting the tolerability of calcifediol. The stable levels achieved in the present study with a weekly dose of 100 µg calcifediol (approximately 40.7 ng/mL) could be considered optimal for clinical purposes. These levels align with recommendations to maintain 25(OH)D concentrations above 30 ng/mL to prevent deficiency-related complications while presenting a lower risk of reaching excessively high levels. Collectively, these findings reinforce that a weekly dose of 100 µg calcifediol represents a safe and effective strategy for correcting vitamin D deficiency, regardless of baseline levels.

Calcifediol supplementation can offer significant health benefits while exhibiting a low risk of toxicity. Our long-term study of more than 401 subjects showed that there is no difference in overall measured adverse effects between weekly administration of placebo or calcifediol 100 µg for one year. A 25(OH)D safety threshold was established at 80 ng/mL (200 nmol/L) in the study, while clinical manifestations are defined that may occur above 100 ng/mL (250 nmol/L) [25] or even 150–200 ng/mL (375–500 nmol/L) [26] and are often associated with hypercalcemia. In this study, only one subject reached values above 100 ng/mL (100.04 ng/mL) at week 52 without experiencing any toxicity or adverse event and with serum calcium levels within the normal range.

It can be highlighted that the main strengths of this study lie in the large sample size, the long follow-up period of up to one year and the performance of all analytical determinations in a central laboratory. As a *post hoc* analysis, it is inherently limited by the design of the original study, including the lack of an active comparator. However, the placebo-controlled designs accounted for seasonal variations, which are crucial in understanding the fluctuations of endogenous 25(OH)D levels compared to the stability achieved with over-the-year calcifediol treatment. Additionally, the higher percentage of women in the study population may represent a limitation, as it might not fully reflect the general population. However, this gender predominance likely corresponds to the higher prevalence of vitamin D deficiency observed among women in the general population. According to Cui et al. (2023) [7], a meta-analysis of 90 studies across 45 countries demonstrated that women are 1.2 times more likely than men to present 25(OH)D levels below 20 ng/mL (50 nmol/L). This disparity aligns with broader epidemiological trends and is likely influenced by the proportion of postmenopausal women included in our study (46.8% of women). Importantly, the proportion of female and male participants was well-balanced across the placebo and calcifediol treatment groups, ensuring comparability of the results. Further research should consider strategies if achieving a more balanced gender distribution is pursued.

In conclusion, the proven safety and efficacy of the weekly formulation of calcifediol 100 µg in patients with a variety of baseline 25(OH)D levels indicate that it could serve as a universal recommendation for correcting vitamin D deficiency. This would reduce the need for dose adjustments based on baseline levels, simplifying clinical decision-making. Patients could also prefer a weekly posology, potentially increasing compliance. Furthermore, a standardized loading and maintenance dosing strategy could enhance treatment adherence and optimize resource utilization in healthcare systems. Additionally, these findings provide valuable evidence that could inform policymakers in the development of population-wide vitamin D supplementation programs.

## Figures and Tables

**Table 1:** Demographics and baseline characteristics of the Safety Population. Number and percentage of subjects in the placebo or calcifediol 100 µg treatment groups.

		Placebo (*n* = 128)	Calcifediol 100 µg (*n* = 266)
Gender	Female	95 (73.08%)	198 (73.06%)
	Male	35 (26.92%)	73 (26.94%)
Race	Black	0 (0%)	4 (1.48%)
	White	127 (96.92%)	260 (73.06%)
	Asian	0 (0%)	3 (1.11%)
	Other (Mixed) *	4 (3.08%)	4 (1.48%)
Age (years)	Mean	51.68	53.02
	Min	18.00	18.00
	Max	77.00	84.00
Height (cm)	Mean	166.53	166.37
	Min	139.00	142.00
	Max	190.00	196.00
Weight (kg)	Mean	79.39	79.05
	Min	46.00	41.60
	Max	149.20	146.00
Abdominal circumference	Mean	95.80	94.89
	Min	64.00	54.00
	Max	142.50	156.00
BMI (kg/m^2^)	Mean	28.57	28.55
	Min	18.70	17.40
	Max	52.50	54.10
BMI subgroup	Under-weighted	0 (0%)	9 (3.36%)
	Normal	53 (41.09%)	77 (28.73%)
	Over-weighted	35 (27.13%)	86 (32.09%)
	Obese	41 (31.78%)	96 (35.82%)

* Other (Mixed) race includes Arabic, Hispanic, Hispano-Latino, Marocco, Magreb and Mulatto.

**Table 2:** Incidence of Bone and Mineral abnormalities at baseline and at week 52. The table indicates the total Safety Population subjects (N), those with available data (n), and the percentage of subjects with low or high levels for the indicated parameters.

		Placebo (N = 128)		Calcifediol 100 µg (N = 266)	
Parameter		Baseline	Week 52	Baseline	Week 52
Alkaline phosphatase	n	128	106	264	238
	Low (%)	0	0.9	1.5	1.7
	High (%)	15.6	12.3	11.4	10.9
Total serum Ca	n	127	106	264	238
	Low (%)	1.6	0	2.7	2.1
	High (%)	0.8	1.9	3.8	4.2
Phosphorous	n	128	106	266	238
	Low (%)	2.3	4.7	1.9	0.4
	High (%)	3.9	1.9	0.4	2.5
Parathyroid hormone	n	124	102	257	263
	Low (%)	0	0	0	0.4
	High (%)	25.8	17.6	20.6	6.5

Normal reference ranges: alkaline phosphatase 40–129 U/L; total serum calcium 8.6–10 mg/dL; phosphorous 0.81–1.45 mmol/L; parathyroid hormone 15–65 pg/mL.

## Data Availability

Data access requests to clinical_rd@faes.es will be possible until 5 years following article publication.

## Supplementary Materials

The following supporting information can be downloaded at: https://www.mdpi.com/article/10.3390/jcm14092976/s1, Figure S1: Mean change from baseline in 25(OH)D levels in the Placebo (grey) and 100 µg calcifediol pool (blue) at each indicated week after treatment initiation. Table S1: Eligibility criteria.
